# Comparison of surveillance trapping methods to monitor *Culicoides* biting midge activity in Trinidad, West Indies

**DOI:** 10.1111/mve.12590

**Published:** 2022-06-15

**Authors:** Tamiko Brown‐Joseph, Christopher A. L. Oura, Christine V. F. Carrington, Lara E. Harrup

**Affiliations:** ^1^ Department of Pre‐Clinical Sciences, Faculty of Medical Sciences The University of the West Indies St. Augustine Trinidad and Tobago; ^2^ Faculty of Medical Sciences, School of Veterinary Medicine The University of the West Indies St. Augustine Trinidad and Tobago; ^3^ Entomology Group The Pirbright Institute Pirbright UK

**Keywords:** CDC light‐suction traps, crepuscular activity, *Culicoides* biting midges, incandescent light, semiochemical lure, surveillance methods, sweep net methods, trap comparisons, Trinidad (W.I.), UV light

## Abstract

*Culicoides* biting midges (Diptera: Ceratopogonidae) are biting nuisances and arbovirus vectors of both public health and veterinary significance in Trinidad. We compared sampling methods to define the behaviour and bionomics of adult *Culicoides* populations at a commercial dairy goat farm. Three static trap designs were compared: (a) Centre for Disease Control (CDC) downdraft UV trap; (b) CDC trap with an incandescent bulb and (c) CDC trap with semiochemical lure consisting of R‐(−)‐1‐octen‐3‐ol and CO_2_ (no bulb). Sweep netting was used to define diel periodicity. A total of 30,701 biting midges were collected using static traps, dominated by female *Culicoides furens* (>70% of trap collections across all three designs). There was no significant difference in the Margalef's index between the three traps; however, trap designs A and C collected a significantly greater number of individuals than trap B, and trap C gained highest species richness. The greatest species richness and abundance of *Culicoides* collected by sweep net was observed between 6:00 and 6:15 pm and notable differences in the crepuscular activity pattern of several species were identified. Comparative data on *Culicoides* species richness, abundance, sex and reproductive status is discussed and can be used to improve surveillance strategies, research designs and risk management.

## Graphical abstract

1. Study on a dairy goat farm compared three static trap designs with (a) UV light; (b) incandescent light or (c) semiochemical lure (no light). Sweep netting was used to define diel periodicity.
2. Trap designs A and C collected significantly greater number of *Culicoides* specimens than trap B; trap C gained highest species richness despite no significant difference found in Margalef's index between traps.
3. Greatest species richness and abundance of *Culicoides* collected by sweep net was observed between 6:00 and 6:15 pm; notable differences in the crepuscular activity pattern of collected species were identified.

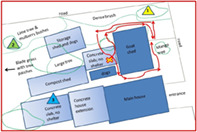

## INTRODUCTION

**FIGURE 1:**
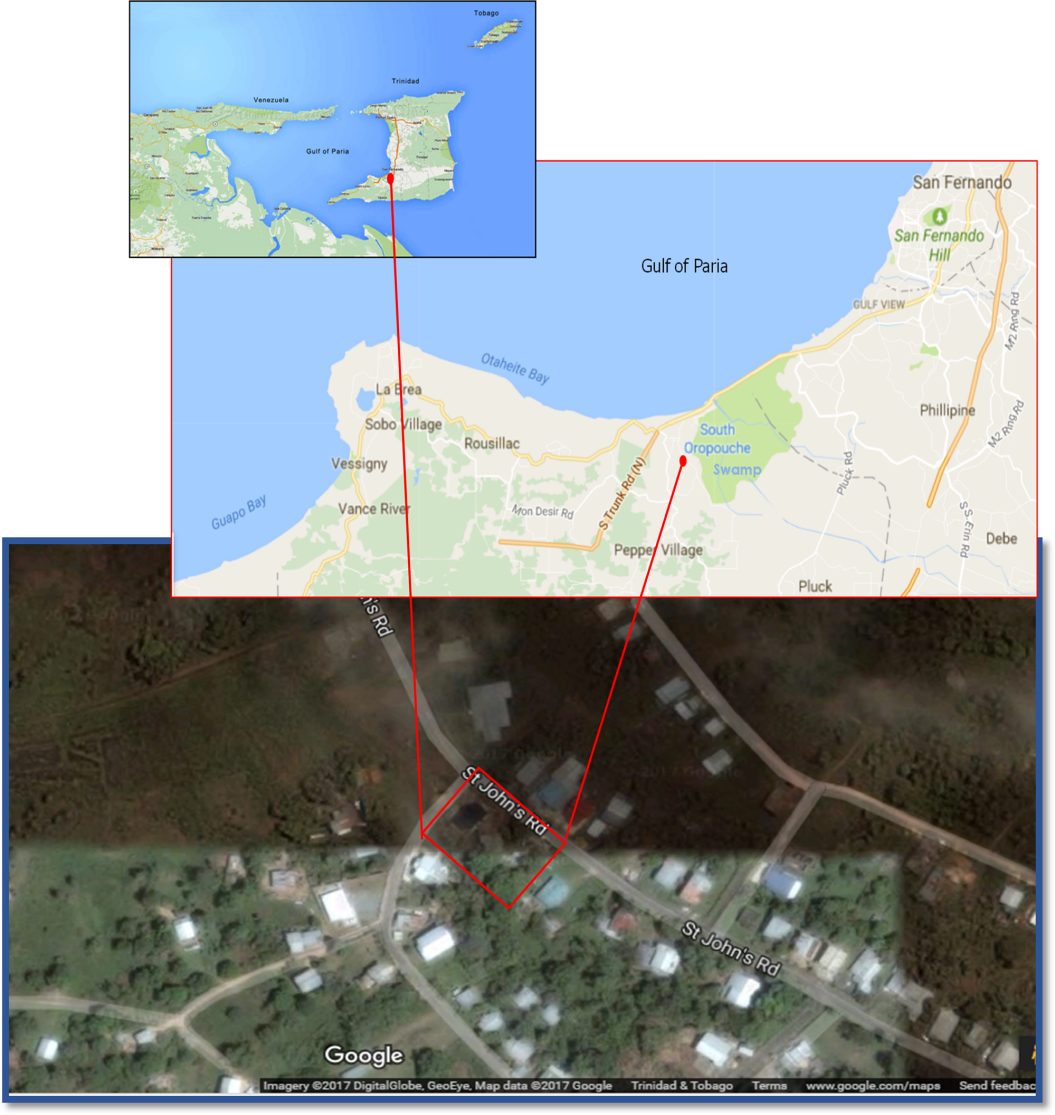
Study site location. Maps show location of Trinidad (and Tobago, W.I.) in relation to the Venezuelan east coast. Red dot indicates location of dairy goat farm in relation to the Gulf of Paria and the South Oropouche swamp. Satellite picture of the small commercial dairy goat farm (red rectangle) showing the surrounding lightly populated rural area located in South Oropouche, Siparia, Trinidad (satellite picture and maps are adapted from Google Imagery 2017 DigitalGlobe, GeoEye, map data).

Trinidad, the larger of the two islands of the Republic of Trinidad and Tobago (T&T), is the southernmost island in the Caribbean archipelago, situated 11 km northeast of the Venezuelan coast (Figure 1). Evidence to date indicates that an abundance and diversity of *Culicoides* biting midges thrive in the hot and humid climate of both islands (Aitken et al., 1975; Greiner et al., 1989; Tikasingh, 1972). In addition, Trinidad's geographical proximity to the South American mainland facilitates the opportunity for wind‐borne introduction of *Culicoides* (Sellers et al., 1978), providing a potential incursion pathway for *Culicoides*‐borne pathogens from South America. There is also current evidence that *Culicoides*‐borne arboviruses are actively circulating in Trinidad (Brown‐Joseph et al., 2017, 2019) and the greater Caribbean region (Anderson et al., 1961; Greiner et al., 1990; Mo et al., 1994; Pinheiro et al., 1981a, 1981b; Tanya et al., 1992).

*Culicoides* biting midges are tiny hematophagous insects approximately 0.5–3.0 mm in length (Carpenter et al., 2015). Over 1400 species of *Culicoides* have been identified globally (Borkent & Dominiak, 2020), 46 of which have been recorded in Trinidad (Aitken et al., 1975; Gumms et al., 1984; Tikasingh, 1972). In the Caribbean, *Culicoides* are biological vectors for viruses that affect animals, such as bluetongue virus (BTV) (Greiner et al., 1990; Mo et al., 1994; Tanya et al., 1992) and epizootic haemorrhagic disease virus (EHDV) (Mo et al., 1994). Brown‐Joseph et al. (2017, 2019) recently determined that BTV serotypes 1, 2, 3, 5, 12 and 17, as well as EHDV‐6 were co‐circulating in Trinidad. In addition to links to arboviruses of veterinary importance, *Culicoides* are also vectors for human pathogens, including Oropouche orthobunyavirus (OROV) (Anderson et al., 1961; Pinheiro et al., 1981a, 1981b) and the parasite *Mansonella ozzardi* (Raccurt, 2018). *Culicoides* species can also be a severe biting nuisance to humans, livestock and equines, which even in the absence of pathogen transmission, can have a significant impact on tourism, forestry and agriculture (Mellor et al., 2000).

Accurately determining the abundance and distribution of vector species of *Culicoides* feeding on relevant hosts is a critical factor in assessing the risk of *Culicoides*‐borne pathogen transmission according to both region and season. Collections of *Culicoides* can be made directly from hosts using a range of approaches including drop‐trapping (Purse et al., 2015), traps attached to hosts (Viennet et al., 2011), or sweep netting (Meiswinkel & Elbers, 2016). These techniques, however, are labour intensive, logistically challenging and are not suitable for large‐scale surveillance activities (Melville et al., 2015). Due to these limitations, a broad range of static and more easily deployable traps have been developed that aim to characterize potential vector species richness and abundance. In surveillance activities, the majority of these traps use attractants such as an artificial light source and/or a semiochemical bait that mimics host odour, combined with a source of suction to collect flying insects.

The diversification in trap designs used for surveying *Culicoides* populations presents a challenge in interpreting abundance and diversity data between regions, countries and research groups (McDermott & Lysyk, 2020). Variation arises both according to the trap design itself, with variation in size, suction, light source and the type of attractant used (Harrup et al., 2012, 2016; Venter et al., 2015). To address this issue, a wide range of studies have examined the comparative effectiveness of different trap designs and attractants in collecting *Culicoides* across the world in Australia (Bishop et al., 2006), Europe (Bray et al., 2020), India (Harrup et al., 2016), North America (Mullens & Gerry, 1998) and South Africa (Venter & Hermanides, 2006; Venter et al., 2009; Scheffer et al., 2012). Systematic comparisons of different attractants using the same trap design platform have been conducted for light wavelength (Bishop et al., 2006; Gonzalez et al., 2016; Harrup et al., 2016; Hope et al., 2015) and semiochemicals (Bhasin et al., 2001; Harrup et al., 2012) and revealed species‐specific responses to these parameters.

Recent studies comparing surveillance approaches for *Culicoides* in the Caribbean and South America are rare, despite the biting midge's noted importance as an arbovirus vector and a biting nuisance in the region. Studies carried out roughly 40 years ago in Trinidad and Tobago used various types of insect traps. A study carried out by Tikasingh (1972) used CDC incandescent light traps and human‐baited sweep net methods to trap biting midges over a period of 14 months to monitor the seasonal abundance and diurnal activity of four *Culicoides* species (*C. diabolicus* Hoffman 1925 (status revised to *C. psuedodiabolicus* Fox 1946, Aitken et al., 1975, *C. foxi* Ortiz 1951, *C. furens* Poey 1853 and *C. pusillus* Lutz 1913). Trapping sites for this study included dense forest where vertical stratification was observed in the predominant species in this study, *C. diabolocus*, trapped mostly in the forest canopy.

A second study carried out by Aitken et al. (1975) set out to identify the *Culicoides spp* present in Trinidad. Incandescent light‐suction traps (both New Jersey and CDC models), sweep nets using human bait, Shannon traps, larval collections and emergence traps designed to collect adult *Culicoides* emerging from larval development material, were used. This study identified 45 *Culicoides* biting midge species by morphology (wing, mesonotum and leg‐band patterns, antennal ratios, papal ratios, proboscis to head ratios, distribution of macrotrichia and sensoria, etc.). These morphological features were extensively documented to generate the biological keys used to identify the *Culicoides* species in this study.

The objectives of this study were (primarily) to compare the trapping capabilities of commercially available CDC light‐suction insect traps fitted with either an ultraviolet (UV) light bulb, incandescent (white) light bulb or a semiochemical lure consisting of R‐(−)‐1‐octen‐3‐ol and carbon dioxide (CO_2_) supplied by sublimating dry ice (light bulb was removed from the CDC light‐suction trap). Their effectiveness in relation to specimen yield, species variety, sex ratios and female reproductive status was measured and compared. Sweep net collections were also compared against the (three) static traps. The secondary aim was to discern the crepuscular activity patterns of the *Culicoides* species that were identified from the sweep net trapping catchments.

## METHODS

### Study location

**FIGURE 2:**
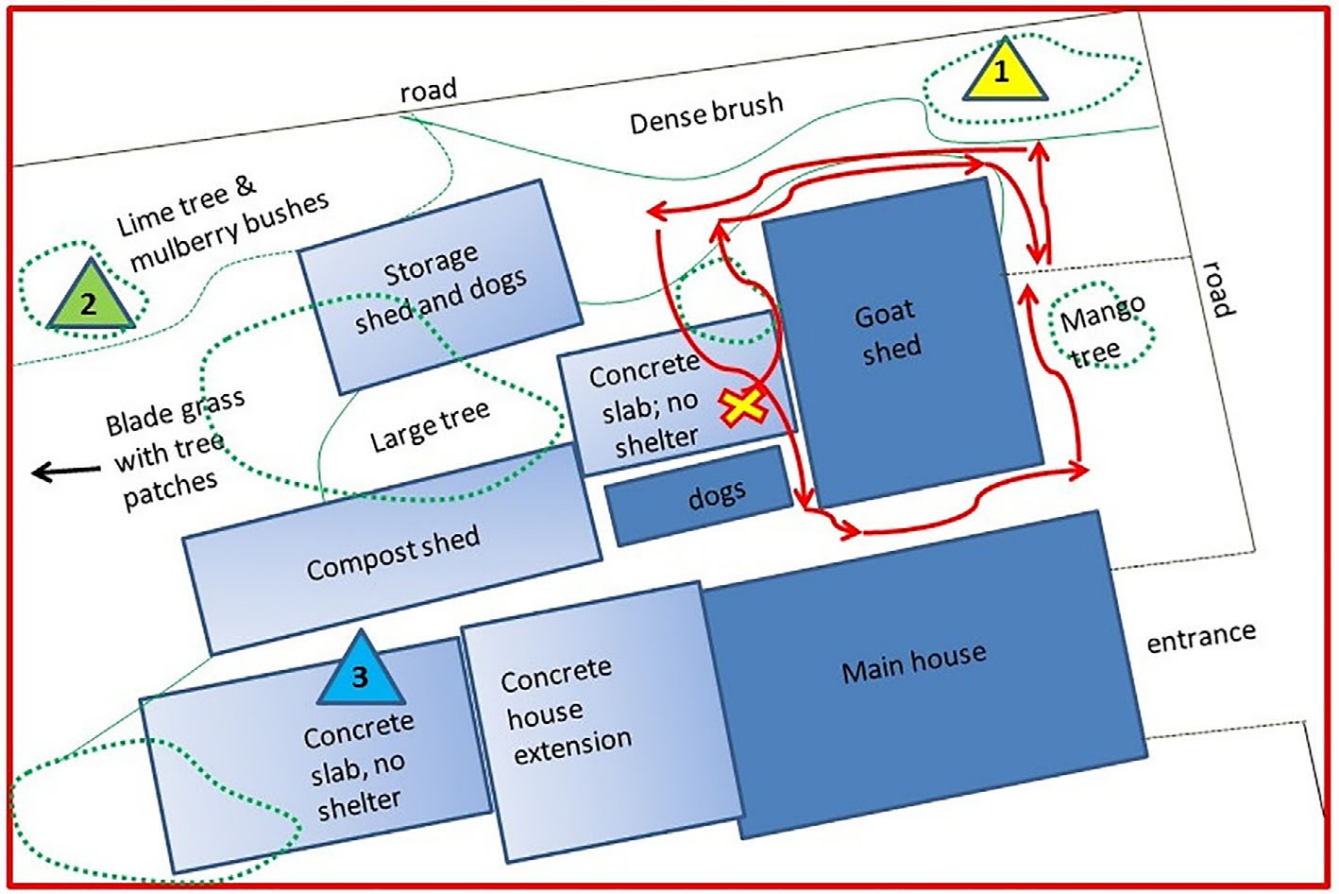
Plan drawing of the dairy goat farm (not drawn to scale) showing the fixed locations (triangles 1, 2 and 3) where each of the three traps were placed overnight for nine nights: *Location 1* was amid dense trees and underbrush; *location 2* was under a lime tree adjacent to cultivated soil and *location 3* was over an exposed concrete slab. The red arrows starting at the yellow X shows the set path taken to sweep net around the goat shed on four separate evenings for the last 2 h of daylight. See Figures S1 and S2 for photos.

This study was conducted at a small commercial dairy goat farm located (approximately 10° 13′ N, −61° 31′ W) in South Oropouche, Siparia, Trinidad, fairly close (<2 km) to the western coastline of the Gulf of Paria and only 0.1 km away from the South Oropouche Swamp (Figure 1). South Oropouche (Siparia district) is approximately 18.5 km south of the densely populated, coastal city of San Fernando. The area is predominantly rural with pockets of moderately populated residential areas and a number of small subsistence and commercial farms. The study site consisted of a small dairy goat farm located within the same compound as the farm owner's family residence. On the farm, 30 penned goats (mixture of Saanen and Anglo‐Nubian breeds [*Capra aegagrus hircus*]) and 12 kenneled guard dogs (common breeds) were held within open air sheds, surrounded by cultivated rows of mulberry bushes (*Morus* spp.), trichantera shrubs (*Trichantera gigantean*), Mulato grass (*Brachiaria* spp) and Guinea grass (*Megathyrsus maximus*) grown to feed the goats (Figures 2, S1 and S2). It is worth noting that the *Jarvis Dairy Goat Farm* is the only ‘no kill’ farm in Trinidad that allows the animals to live out their natural lives on the farm.

### Trap attractants

Three commercially available insect traps with different attractants were compared: (Trap A) Centre for Disease Control (CDC) downdraft UV trap (model 912, John W. Hock Co., Gainesville, FL); (Trap B) CDC trap with incandescent (white)‐light bulb (model 512, John W. Hock Co.); and (Trap C) a CDC trap (model 512, John W. Hock Co.) with the light bulb removed and an attached semiochemical lure consisting of R‐(−)‐1‐octen‐3‐ol and CO_2_ (derived from sublimating dry ice). The CDC light traps (models 512 and 912, John W. Hock Co.) used were modified to enable the wet‐catch of *Culicoides* biting midges by placing a container with dilute soap solution (~200 ml of distilled water with two to three drops of liquid dish soap added) at the bottom of the trap. Both traps were operated using a single 6‐V/12‐A rechargeable battery.

Trapping was carried out overnight, during the dry season, for nine nights (three consecutive nights for three consecutive weeks) from the last week of January to mid‐February (2017). The three fixed locations utilized within the goat farm were between 46 and 61 m apart and were between 15 and 31 m away from the livestock shed containing 30 penned goats and housing four of the kenneled guard dogs. The placement of the different traps (A, B and C) in the different positions (locations 1, 2 and 3) was determined by a 3 × 3 randomized Latin square study design. Each trap was out of the line‐of‐sight of the other two traps and placed approximately 1.2 m above the ground (Figure 2).

Location 1 was above thick vegetation with the traps hung from the branch of a tree with dense foliage, amidst high grass and above damp leaf litter. Location 2 was sited close to cultivated land with the trap hung from a citrus tree (no flowers or fruits present) above exposed loamy, topsoil, adjacent to cultivated agricultural beds with little to no leaf litter. Location 3 was above a concrete slab extension behind the main residence with no soil or plants in the immediate vicinity, although an adjacent open shed housed a compost heap and two of the kenneled dogs (Figure 2). All traps were positioned and operational from 1 h before sunset (between 5:15 and 5:45 pm) until 2–3 h after sunrise (8:30–9:30 am). Arthropods were collected into water containing a drop of non‐bleaching detergent. At the end of each trapping period, trapped arthropods were rinsed with distilled water and transferred into 70% (vol/vol) ethanol for storage.

### *Comparison of crepuscular* Culicoides *activity by sweep net collections*

Sweep net collections were conducted separately at the goat farm during the middle of the dry season, for a total of four evenings between mid‐March to mid‐April (2017). The days selected for sweep net collections had little to no wind and no rain during the day. On each day, for the final 2 h of daylight (4:15–6:15 pm), flying insects were collected using a sweep net every 15 min for a total of 5 min per sweep net collection, following a set path (Figure 1) around the open‐air shed containing 30 penned goats and four (of the 12) kenneled guard dogs. After each sweep net session, the net was placed on dry ice for 5 min to euthanize any collected insects, which were then removed from the net with an aspirator and transferred to sterile, labelled specimen cups containing 70% (vol/vol) ethanol prior to further analysis.

### *Identification of* Culicoides *specimens*

For samples collected using the traps and the sweep net, *Culicoides* were separated from other arthropods (bycatch) and identified, where possible, to species level, by morphology using a biological identification key (Aitken et al., 1975). Specimens of the morphologically cryptic subgenus *Hoffmania* Fox that were not discernable to their exact group (*hylas* or *guttatus*) or species were identified to subgenus level only. The species, sex and reproductive status for female specimens (nulliparous/non‐pigmented; blood fed; gravid; parous/pigmented) were recorded for all *Culicoides* collected.

### *Comparison of* Culicoides *species richness with respect to trap attractant and location*

The number of *Culicoides* species (i.e., the species richness/variety) collected by the three trap types, was compared using Margalef's index, such that *Margalef's index* = *(S* − *1)*/*ln N*, where *S* is the total number of species collected in a sample (i.e., one trap collection), *N* is the total number of individuals in the sample and *ln* is the natural logarithm (Margalef, 1958). Margalef's index was also calculated to compare the species richness among the three different environments found at the three set positions (locations 1–3).

### Statistics and data visualization

All data visualization was done using R v. 3.5.1 (R Development Core Team, 2018) with RStudio v. 1.1.453 (RStudio Team, 2018). Data were visualized using the ggplot2 v3.0.0 (Wickham, 2016) package. All *Culicoides* abundance data were log transformed (log(1 + *x*)^10^) to enable visualization; however, for model fitting, the raw abundance data were rescaled to create a data range between 0 and 1 using the ‘scales’ v.0.5.0 package (Wickham, 2017).

To investigate the effect of trap type on the number of *Culicoides* and species distribution, generalized linear mixed models (GLMM) with a binomial error distribution and a logit link function were implemented in a Bayesian setting using the bglmer function in package ‘blme’ v. 1.0‐2 (Dorie, 2014). The GLMMs were fitted by maximum likelihood with the Laplace approximation with flat covariance priors and normal fixed priors, with *day of collection* included as a random effect, *trap type* and *trap location* considered as potential additional fixed predicator. Final models were obtained using a backwards‐stepwise‐selection‐based procedure (Zeileis et al., 2008), such that variables that did not contribute significantly to explaining variation in trap catch were successively eliminated on the basis of the Bayesian information criterion (BIC) (Schwarz, 1978) until the removal of a variable caused an increase in BIC of two or more. Differences in trap catch size between trap types were then assessed using multiple Tukey's all‐pair comparisons using the ‘glht’ function in package multcomp version 1.4‐8 (Hothorn et al., 2008).

In addition, linear and polynomial regression models in ‘stats’ v. 3.5.0 package (R Development Core Team, 2018) were utilized to visualize the activity profile of *Culicoides* and select *Culicoides* species collected by sweep netting with *time of collection* or a quadratic function of *time of collection* considered as a fixed predictor. Both Akaike information criterion (AIC) (Akaike, 1973) using function ‘AIC’ in the ‘stats’ v. 3.5.0 package (R Development Core Team, 2018) and Wald tests, using function ‘wald test’ in the lm test v.0.9‐36 package (Zeileis & Hothorn, 2002), were utilized to assess model fit with and without a quadratic term.

## RESULTS

### Comparison of trap attractants

**FIGURE 3:**
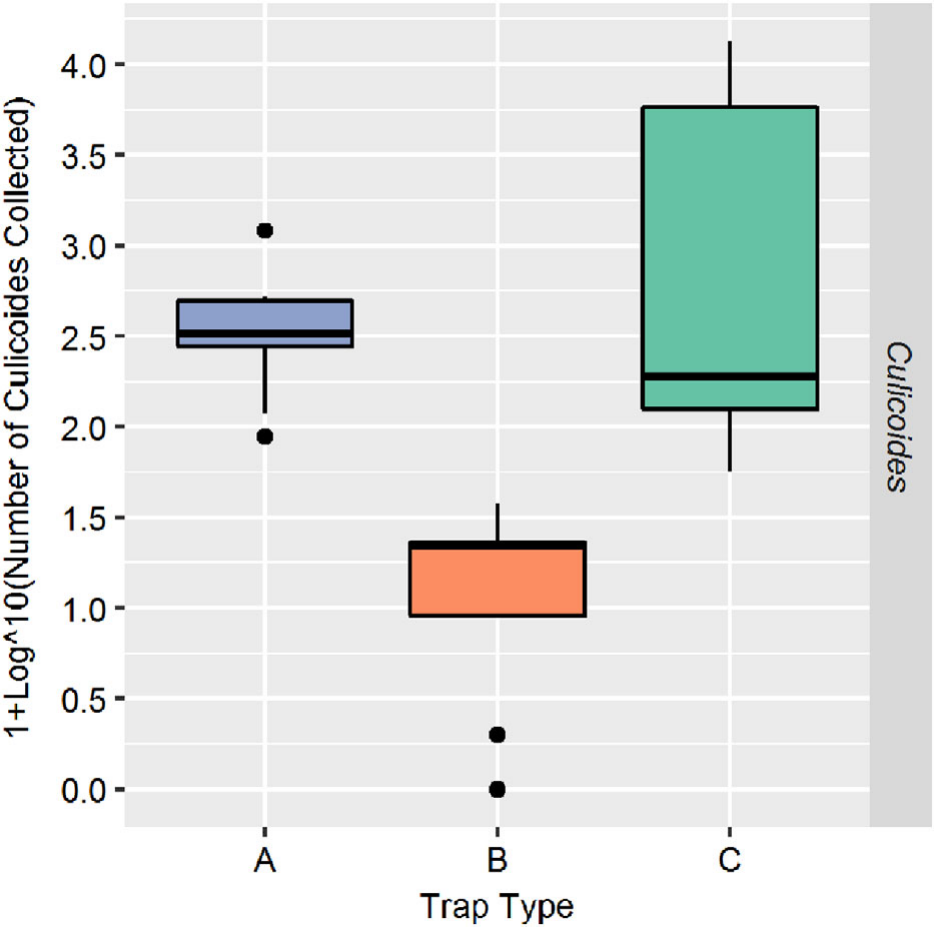
Box‐and‐whisker plots illustrating the total number of *Culicoides* collected by each trap type (A [blue]: Centre for Disease Control [CDC] downdraft UV trap [model 912, John W. Hopkins Co.]; B [red]: CDC trap with incandescent [white]‐light bulb [model 512, John W. Hopkins Co.]; C [green]: unlit CDC trap [model 512, John W. Hopkins Co.] with a semiochemical lure consisting of R‐(−)‐1‐octen‐3‐ol and CO_2_ [from sublimating dry ice]) over nine nights of trapping

A total of 30,701 *Culicoides* specimens were collected over the nine nights of trapping (Table 1). The majority of *Culicoides* (*n* = 26,722; 87.04%) were collected by trap C (semiochemical lure [R‐(−)‐1‐octen‐3‐ol) with CO_2_]). Trap A (UV light) collected 3823 *Culicoides* (12.45%), whereas trap B (incandescent light) collected a total of 157 *Culicoides* (0.51%). The number of *Culicoides* collected by traps A and B were fairly consistent in specimen yield between each of the nine nights of trapping. Trap C had the greatest variability in the number of *Culicoides* collected per trap night (Figure 3), with a significantly skewed distribution in the number of specimens collected over the nine nights (range: 70 > *n* < 13,438). The vast majority (*n* = 13,438) of the *Culicoides* were collected by trap C on night six; the second highest amount (*n* = 6698) was collected on night one (Table 1).

**TABLE 1:** Total number of *Culicoides* biting midges collected (male; female) by each trap over nine nights of trapping

Collection night	A: UV light	B: White light	C: 1‐octen‐3‐ol/CO_2_
1	488 (99; 389)	22 (6; 16)	6698 (17; 6681)
2	118 (58; 60)	35 (7; 28)	134 (0; 134)
3	325 (48; 281)	8 (3; 5)	189 (2; 187)
4	524 (89; 433)	11 (4; 7)	70 (0; 70)
5	277 (173; 104)	21 (2; 19)	56 (0; 56)
6	1211 (380; 831)	37 (5; 32)	13438 (8; 13430)
7	87 (31; 56)	1 (0, 1)	216 (2; 214)
8	495 (135; 361)	1 (0; 1)	124 (2; 122)
9	296 (34; 262)	21 (6; 15)	5797 (0; 5797)
Total	3823 (1043; 2779)	157 (33; 124)	26722 (31; 26691)

*Note*: Trap A: Centre for Disease Control (CDC) downdraft UV trap. Trap B: CDC incandescent (white)‐light downdraft trap. Trap C: unlit CDC downdraft trap with a semiochemical lure consisting of R‐(−)‐1‐octen‐3‐ol and CO_2_. Colour of cell indicates the set location on the farm that the trap was placed (Figure 2) according to a 3 × 3 randomized Latin square design for nine nights of trapping (yellow = position 1 [dense brush]; green = position 2 [lime tree] and blue = position 3 [concrete slab]).

Female *Culicoides* dominated trap collections (96.33%; *n* = 29,594), with a ratio of 26.73 collected for every male *Culicoides*. Of the *Culicoides* females collected, 9.39% (*n* = 2779), 0.42% (*n* = 124) and 90.19% (*n* = 26,691) were collected by traps A, B and C, respectively (Table 1). Very few gravid (<< 0.01%; *n* = 2) and blood‐fed (0.24%; *n* = 70) females were collected by the three trap types. More non‐pigmented (nulliparous) female *Culicoides* (75.84%; *n* = 22,442) were collected than pigmented (parous) (22.30%; *n* = 6958) (Figure S3). The reproductive status of a small proportion (1.63%; *n* = 481) of the female *Culicoides* collected could not be conclusively determined as either pigmented or non‐pigmented due to their very dark body colour obscuring the potential presence of the relevant cherry‐red abdominal pigmentation pattern (Dyce, 1969). Of the 1107 male *Culicoides* specimens collected, the vast majority of males (94.22%; *n* = 1043) were collected by trap A (UV‐light‐suction) with traps B (incandescent light) and C (semiochemical lure) capturing 2.98% (*n* = 33) and 2.80% (*n* = 31) of the males respectively (Table 1). It is also notable that the mechanical sweep net method yielded a relatively high proportion of males (9.93%; *n* = 95) when compared to the static traps (Table 4).

**FIGURE 4:**
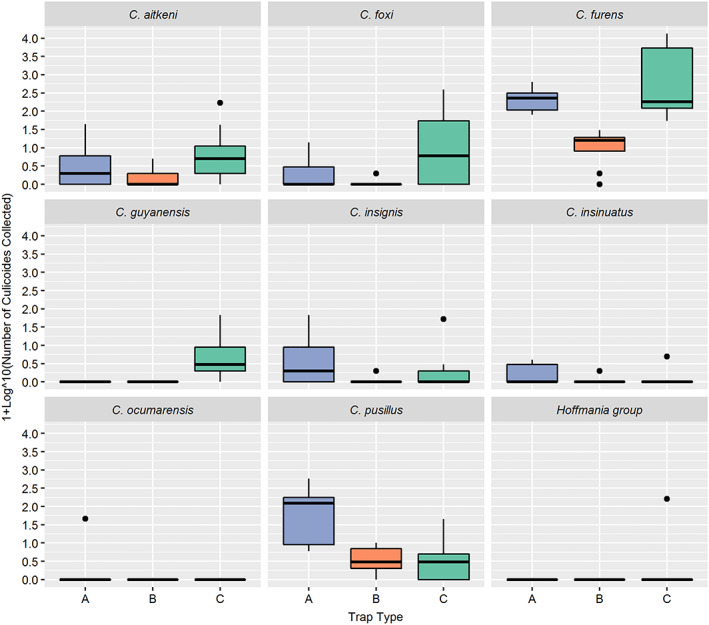
Box‐and‐whisker plots illustrating the number of *Culicoides* (by species) collected for each trap type (A [blue]: Centre for Disease Control [CDC] downdraft UV trap [model 912, John W. Hopkins Co.]; B [red]: CDC trap with incandescent [white]‐light bulb [model 512, John W. Hopkins Co.]; C [green]: unlit CDC trap [model 512, John W. Hopkins Co.] with a semiochemical lure consisting of R‐(−)‐1‐octen‐3‐ol and CO_2_ [from sublimating dry ice]) over nine nights of trapping

In addition to morphologically cryptic specimens from the subgenus *Hoffmania*, a total of eight *Culicoides* species were collected and identified: *C. aitkeni* Wirth and Blanton, 1968, *C. foxi* Ortiz, 1950, *C. furens* (Poey), 1853, *C. guyanensis* Floch and Abonnenc, 1942, *C. insignis* Lutz, 1913, *C. insinuatus* Ortiz and León, 1955, *C. ocumarensis* Ortiz, 1950, *C. pusillus* Lutz, 1913 (Table 2). *Culicoides furens* dominated collections representing 91.0% (*n* = 27, 923) of all *Culicoides* collected. Of these, 91.6% of the *C. furens* were collected by trap type C which utilized the semiochemical lure (R)‐(−)‐1‐octen‐3‐ol with CO_2_. Five of the species (*C. aitkeni*, *C. foxi*, *C. furens*, *C. insinuatus* and *C. pusillus*) were collected in all three trap types (Figure 4). However, *C. guyanensis* (*n* = 105) and the subgenus *Hoffmania* specimens (*n* = 162) were found only in collections from trap type C, whereas *C. ocumarensis* (*n* = 45) was found only in collections from trap type A, which utilized UV light as an attractant (Figure 4). Species richness of the trap collections, as indicated by Margalef's index (Table 2), showed no significant difference in the species richness collected by each of the three trap types despite the significant difference in the total number of *Culicoides* collected by each of the trap types.

**TABLE 2:** Total number of *Culicoides* biting midges collected (male; female) by species for each trap over nine nights

*Culicoides* species	Trap type		
	A	B	C
*C. aitkeni*	56 (4; 52)	8 (0; 8)	233 (2; 231)
*C. foxi*	19 (4, 15)	1 (0; 1)	516 (2; 514)
*C. furens*	2238 (704; 1534)	113 (29; 84)	25,572 (23; 25,549)
*C. guyanensis*	0 (0; 0)	0 (0; 0)	105 (0; 105)
*C. insignis*	107 (4; 103)	0 (0; 0)	55 (0; 55)
*C. insinuatus*	8 (0; 8)	1 (1; 0)	8 (0; 8)
*C. ocumarensis*	45 (2; 43)	0 (0; 0)	0 (0; 0)
*C. pusillus*	1346 (325; 1021)	32 (3; 29)	69 (4; 65)
Subgenus *Hoffmania*	0 (0; 0)	0 (0; 0)	162 (0; 162)
Number of species collected (S)	7	5	8
Total number collected (N)	3819	155	26,720
Margalef's index = (*S* − 1)/*ln N*	0.727	0.793	0.687

*Note*: Trap A: Centre for Disease Control (CDC) downdraft UV trap. Trap B: CDC incandescent (white)‐light trap. Trap C: unlit CDC trap with a semiochemical lure consisting of R‐(−)‐1‐octen‐3‐ol and CO_2_. Species richness of the three traps was assessed using *Margalef's index* = (*S* − 1)/*ln N*, where *S* is the total number of species collected by the trap, *N* is the total number of individuals collected and *ln* is the natural logarithm (Margalef, 1958).

In total, 8.2% (*n* = 2516), 86.1% (*n* = 26,435) and 5.7% (*n* = 1750) *Culicoides* specimens were collected in locations 1–3, respectively (Table 3). The majority of *Culicoides* collected by trap A (53.2%, *n* = 2032) were collected amid the lush vegetation (location 1). *Culicoides* collected by trap B specimens were more evenly split between the lush vegetation (location 1) (40.1%, *n* = 64) and the concrete environment (location 3) (46.5%, *n* = 73). However, in contrast, trap C collected the most *Culicoides* (97%, n = 25,933) (Table 3) in the cultivated area on the farm (location 2) (Figure 1).

**TABLE 3:** Total *Culicoides* biting midges (species) collected over nine nights of trapping during the dry season with respect to the location traps were placed on a goat farm (1: tree amid lush vegetation; 2: tree amid cultivated land and 3: steel equipment on concrete slab) and the type of trap used (A: Centre for Disease Control [CDC] downdraft UV trap; B: CDC incandescent [white]‐light trap; C: unlit CDC trap with a semiochemical lure consisting of R‐(−)‐1‐octen‐3‐ol and CO_2_).

Trap type/species	Location			Total
	1	2	3	
A	2032	482	1308	3822
B	64	20	73	157
C	402	25,933	369	26,722
*C. aitkeni*	20 (C)	219 (C)	58 (A)	297
*C. foxi*	12 (C)	502 (C)	22 (A)	536
*C. furens*	1481 (A)	25,300 (C)	1143 (A)	27,924
*C. guyanensis*	3 (C)	96 (C)	6 (C)	105
*C. insignis*	1 (C)	58 (C)	106 (A)	165
*C. insinuatus*	2 (A)	8 (C)	7 (A)	17
*C. ocumarensis*	‐	‐	45 (A)	45
*C. pusillus*	997 (A)	89 (C)	363 (A)	1449
Subgenus *Hoffmania*	‐	162 (C)	‐	162
Totals	2516	26,435	1750	30,701
Males: females	575: 1941	294: 26,141	238: 1512	
Margalef's index	0.766	0.687	0.937	

*Note*: Total males: females caught in each location are also presented. The trap type that collected the majority of each species at the respective location is in parentheses. Species richness of the three locations was assessed using Margalef's index = (*S* − 1)/*ln N*, where *S* is the total number of species collected at the location, *N* is the total number of individuals collected there and *ln* is the natural logarithm (Margalef, 1958).

Another noteworthy observation was made regarding the relative proportions of non‐*Culicoides* (bycatch) versus *Culicoides* specimens collected by the different traps A, B and C (actual values not recorded). Trap A (UV light) consistently caught much larger (unsorted) collections than trap B (incandescent light), but always had a significantly higher proportion (≥50%) of bycatch when compared to the collections from trap C (semiochemical lure [(R)‐(−)‐1‐octen‐3‐ol/CO_2_]), which was very specific in collecting female *Culicoides* exclusively.

### *Comparison of crepuscular* Culicoides *activity by sweep net collection*

**FIGURE 5:**
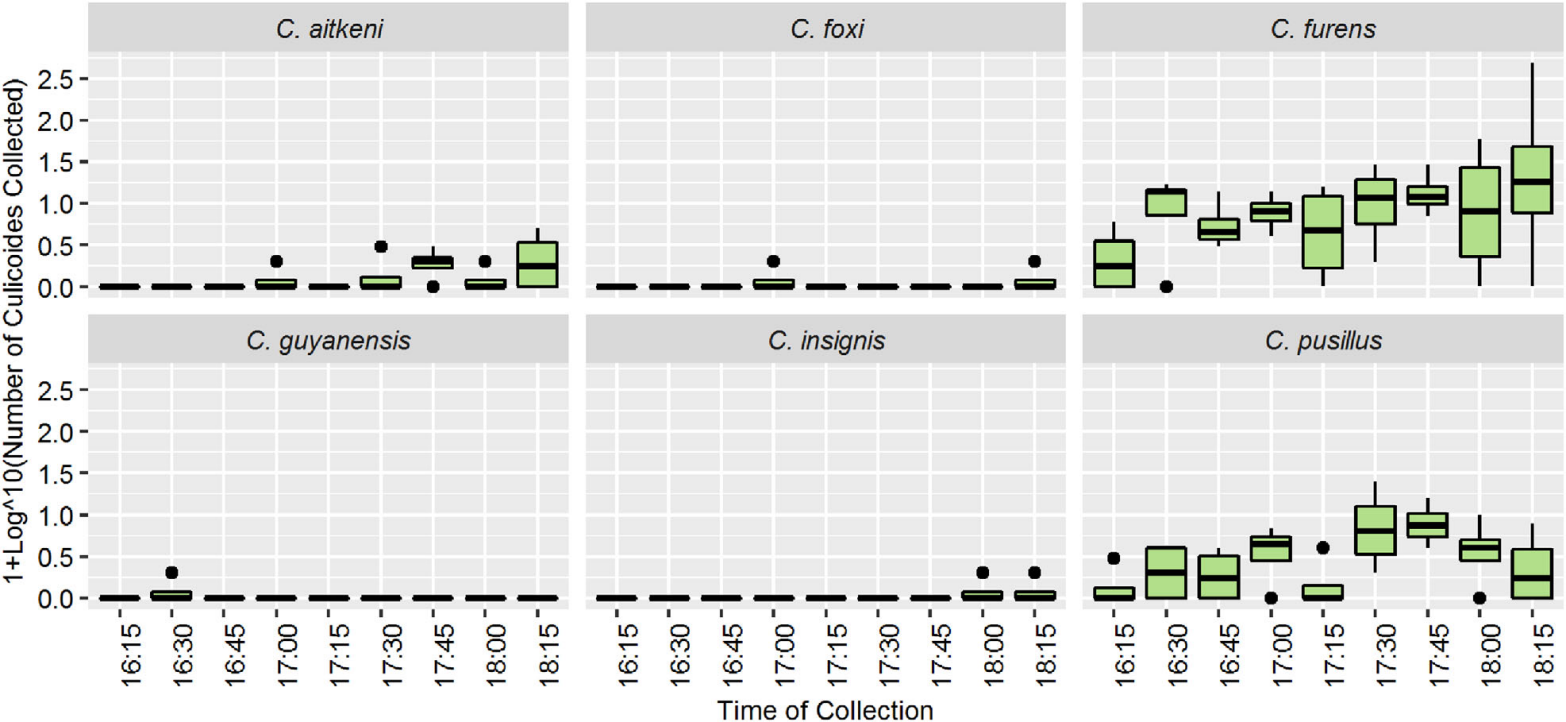
Box‐and‐whisker plots of the number of *Culicoides* (by species) collected by sweep net trapping. Sweep net trapping was conducted in 15‐min time intervals during the last 2 h of daylight along a fixed path at a goat farm for a total of four nights (South Oropouche, Trinidad, W.I).

A total of 975 *Culicoides* specimens were collected by sweep net over a 4‐day period during the final 2 h of daylight (4:15–6:15 pm). In total, six species of *Culicoides* were collected, (*C. aitkeni*, *C. foxi, C. furens, C. guyanensis, C. insignis* and *C. pusillus*) (Figure 5). Collections were dominated by three of these species with *C. furens*, *C. pusillus* and *C. aitkeni* making up 85.44% (*n* = 833), 12.62% (*n* = 123) and 1.44% (*n* = 14) of the total collections, respectively. The three remaining species, *C. foxi* (0.21%; *n* = 2), *C. insignis* (0.21%; *n* = 2) and *C. guyanensis* (0.1%; *n* = 1) were each collected in only one of the sweep net sessions on different evenings (Figure 5; Table S4).

Female *Culicoides* also dominated the sweep net collections (90.15%; *n* = 879) where the majority (83%; *n* = 730) were non‐pigmented, 9.7% (*n* = 85) were pigmented and 5.8% (*n* = 51) were blood fed. The reproductive status of the remaining 1.5% (*n* = 13) was undetermined. No gravid females were collected using the sweep net method. However, it was noted that the relative proportion of blood‐fed females collected using the sweep net (5.8%; 51 out of 879 total) was ~23 times higher than the relative proportion of blood‐fed females (0.25%; *n* = 72) collected by all three traps with attractants (A, B and C) combined (*n* = 29,217) (Figure S3).

**FIGURE 6:**
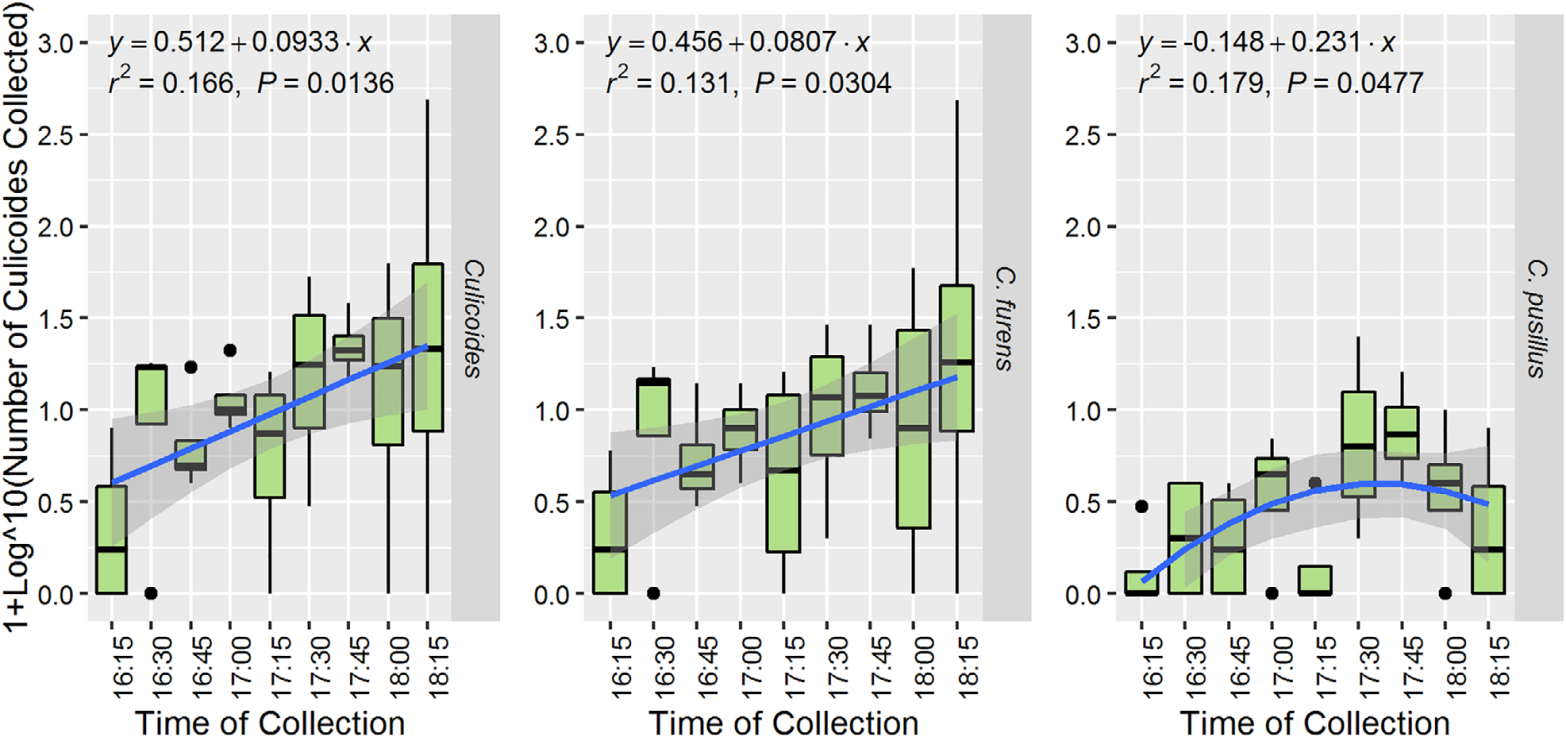
Box‐and‐whisker plots illustrating the crepuscular activity profile for all *Culicoides* and for the two dominant species (*C. furens* (Poey), 1853 and *C. pusillus* Lutz, 1913) collected by sweep net. Sweep net trapping was conducted in 15‐min time intervals during the last 2 h of daylight along a fixed path at a goat farm for a total of four nights (South Oropouche, Trinidad, W.I.).

Linear and polynomial regression analyses revealed that the mean number of *Culicoides* collected using the sweep net increased as daylight intensity decreased with time (Figure 6). No significant differences were observed between the numbers of males and females active at each time point (Table 4). The number of *Culicoides* species (i.e., species richness) collected during each sweep net collection interval ranged from two to four, with *C. furens* and *C. pusillus* present throughout the entire 2‐h collection periods. The highest species richness (four different species) occurred between 6:00 and 6:15 pm (Figure S3). Of the two species that dominated the sweep net collections, *C. pusillus* activity gradually increased and peaked at 5:30 pm, whereas *C. furens* activity increased continually with the approaching sunset up to 6.15 pm (Figure 5). It was also noted that *C. aitkeni* consistently appeared from 5:00 pm onwards on all four nights of trapping despite trapping starting at 4:15 pm (Figure 4). The overall Margalef's index for the sweep net method was 0.872, which indicated that the species richness (Margalef's index) derived from this mechanical trapping method was notably higher than that for traps A, B and C with attractants (Table 2).

**TABLE 4:** Total number of *Culicoides* collected (male; female) by time point using sweep net trapping for four evenings

Collection time point	Collection night			
	1	2	3	4
4:15 pm	2 (1; 1)	‐	7 (4; 3)	‐
4:30 pm	0	16 (8; 8)	16 (6; 10)	16 (5; 11)
4:45 pm	3 (1; 2)	4 (0; 4)	4 (1; 3)	16 (2;14)
5:00 pm	7 (1; 6)	9 (3; 6)	6 (0; 6)	19 (0; 19)
5:15 pm	4 (0; 4)	‐	10 (3; 7)	17 (1; 16)
5:30 pm	2 (0; 2)	10 (3; 7)	27 (9; 18)	52 (3;49)
5:45 pm	21 (4; 17)	14 (8; 6)	19 (7; 12)	37 (2; 35)
6:00 pm	11 (0; 11)	24 (12; 12)	62 (6; 56)	‐
6:15 pm	30 (0; 30)	14 (5; 9)	3 (0; 3)	483 (0; 483)
Total	80 (7; 73)	91 (39; 52)	154 (36; 118)	640 (13; 627)

## DISCUSSION

This study compared the effectiveness of three static traps using different wavelengths of light (UV vs. incandescent light) and a semiochemical lure ((R)‐(−)‐1‐octen‐3‐ol/CO_2_) as attractants. The surveillance (trapping) capabilities of the static traps were compared to a mechanical (sweep net) trapping method in relation to *Culicoides* specimen yield, sex, reproductive status and species richness, unlike in earlier trapping studies carried out in Trinidad (Aitken et al., 1975; Tikasingh, 1972).

### Trap efficiency and feasibility for surveillance activities

Traps A (UV light) and C (semiochemical lure [(R)‐(−)‐1‐octen‐3‐ol/CO_2_]) were significantly more efficient than trap B (incandescent light) in catching large numbers of *Culicoides*, collecting as much as 25 and 174 times more *Culicoides* specimens respectively than trap B. This observation is consistent with previous studies that compared incandescent to UV light traps (Venter et al., 2006) and UV light traps to semiochemical traps (Harrup et al., 2012). It should however be noted that the number of specimens collected was skewed by the high numbers of *C. furens* caught with the semiochemical trap (C) located near cultivated mulberry bush beds. *Culicoides furens* is not a known vector species, although it is responsible for high levels of nuisance biting. However, *C. furens* is an autogenous species with a very wide host range, which may explain why it was present in such high numbers on the farm.

The observation that traps A (UV light) and B (incandescent light) had relatively high proportions of (non‐*Culicoides*) bycatch, whereas trap C (semiochemical lure [(R)‐(−)‐1‐octen‐3‐ol/CO_2_]) exclusively trapped *Culicoides* females was not surprising since haematophagous *Culicoides* locate their hosts primarily through the olfaction and the detection of body heat (Harrup et al., 2012; Mands et al., 2004; Scheffer et al., 2012; Venter et al., 2011). High proportions of bycatch in collections, as seen with the light‐baited traps (A and B), will impact the time and labour requirements when planning *Culicoides* trapping. Therefore, using traps with very little to no bycatch, like trap C (semiochemical lure [(R)‐(−)‐1‐octen‐3‐ol/CO_2_]), will aid to minimize the potential drain on restricted labour resources. This benefit may however be offset by the high cost and impermanence of semiochemical lures, so this will not be suited to projects/studies with relatively long timelines and/or low budgets, as is the case with most *Culicoides* surveillance programmes.

Male *Culicoides* represented 3.61% of the total collection from all three static traps. It is noteworthy that the vast majority (94.22%) of all the males were caught with the UV light attractant (trap A), and just over half (51.94%) of the males were caught in the location where there was lush vegetation under a tree. Based on this data, it is recommended that studies targeting male *Culicoides* should use UV light‐suction traps placed in areas with underbrush.

Two of the *Culicoides* species collected showed apparent biases to particular attractants: *C. ocumarensis* was exclusively collected by trap A (UV light) whereas *C. guyanensis* was exclusively collected by trap C (semiochemical lure [(R)‐(−)‐1‐octen‐3‐ol/CO_2_]). This observation indicates the importance of utilizing both semiochemical lures as well as UV light traps wherever possible to ensure that no *Culicoides spp*. are excluded when trapping for species identification and taxonomical studies. The data also shows that utilizing incandescent light traps (B) alone for routine surveillance for vector or nuisance species may prove disadvantageous since trap B yielded the least number of specimens, and trapped two to three less species than traps A and B respectively, and failed to trap a known BTV vector species, *C. insignis*.

Blood‐meal analysis studies require the trapping of as many blood‐fed *Culicoides* specimens as possible. However, blood‐fed females are satiated and unlikely to be attracted to light traps and traps with semiochemical lures that may mimic hosts. This may explain why relatively low number of blood‐fed females were caught by the three static traps (A, B and C) overall. Results showed the sweep net method caught the highest (relative) proportion of blood‐fed females when compared to that of all three static traps, although these results need to be interpreted with caution since the numbers of blood‐fed individual caught by the three traps were small.

### Trap collections: species richness

All *Culicoides* specimens were morphologically identified to their species level where possible; including *C. aitkeni* Wirth & Blanton 1968 (of the hylas Group); *C. foxi* Ortiz 1951, *C. ocumarensis* Ortiz 1950 (aka *C. filariferus* Hoffman 1939 status revised; Aitken et al., 1975) and *C. insignis* Lutz 1913 (of the guttatus Group—these species are found within subgenus *Hoffmania* Fox. Some adult females were identified to be in this subgenus, but it was difficult to morphologically discern their species either due to (i) the genetically cryptic nature of the subgenus *Hoffmania* groups or (ii) their very dark pigmentation if in a parous/pigmented reproductive state. These particular specimens were identified to the subgenus *Hoffmania* level only.

Although there was a significant difference in the efficiency of the traps with respect to *Culicoides* specimen yield, no significant difference in Margalef's species richness index (i.e., species richness) was observed between the collections from the three static traps and those collected via sweep net. Trap B collected the least number of species (5), followed by Trap A and C with 7 and 8 species trapped, respectively. However, the UV light‐suction trap (trap A) may be the most appropriate trap to use, as it combines high species richness with a relatively smaller collection size, reducing the time and costs required to sort through the collections. Overall, eight *Culicoides* species were collected in this study, including representatives of the morphologically cryptic *Hoffmania* subgenus.

*Culicoides furens*, which is primarily known to breed in salt marshes and mangrove swamps (Tikasingh, 1972), dominated trap collections (90.9%) and sweep net collections at the same site (88%), which is consistent with previous studies conducted in Trinidad (Tikasingh, 1972) and in Southeast USA (Breidenbaugh et al., 2009). The predominance of *C. furens* on the farm is most likely due to the proximity of the brackish marshland, only 0.1 km away, a favourable habitat for this species. The adjacent South Oropouche Swamp is located where the freshwater of the South Oropouche River mixes with saltwater as it empties into the Gulf of Paria. Although *C. furens* is not a vector species for any known viruses or parasites (Greiner et al., 1992), it is well documented as a nuisance species in several tourism‐dependent countries (Aitken et al., 1975) and was indeed a source of distress to the animals on the farm.

*Culicoides guyanensis* is also known to breed in salt‐water marshes (Aitken et al., 1975); however, its abundance was very low in the trap collections by contrast to *C. furens*. Although *C. guyanensis* has been collected throughout the year in Trinidad (Brown‐Joseph et al., unpublished data), the peak abundance for this species is at the start of the rainy season in May, by contrast to *C. furens* which is most abundant in the dry season starting in February, when this study was conducted (Aitken et al., 1975).

Tikasingh (1972) also found *C. pusillus* to be primarily present in the rainy season, with peak abundance from June to August, which may explain its relative low abundance during this study. There is little to no information on the relative seasonality of the other species collected in this study (*C. aitkeni*, *C. foxi*, *C. insignis*, *C. insinuatus* and *C. ocumarensis*) and how this may have impacted their relative abundance.

### *Identification of* Culicoides *vector species*

The identification of vector species found in a particular location/region is of high importance when conducting surveillance studies. In order to gain a full understanding of the epidemiology of vector‐borne disease and to aid in predicting timelines for potential outbreaks, it is important to have a full understanding of the preferred habitat and seasonal abundance throughout the year of the vector species in question.

Although *C. furens* and *C. guyanensis* are not considered vector species, *C. insignis* and *C. pusillus* are. It has been established that *C. insignis* is a competent vector for both BTV (Tanya et al., 1992) and EHDV (McGregor et al., 2021). BTV (serotype 3) has also been isolated from *C. pusillus* females in Jamaica; identifying *C. pusillus* as a potential vector species for BTV (Tanya et al., 1992). So, despite the low numbers trapped in this study, two vector species for BTV and EHDV were identified on the goat farm, indicating a possible route of transmission for these two viruses among the goats on the farm. Confirmation of this via group‐specific real‐time PCR for BTV and EHDV was outside the scope of this particular study.

### Trap collections: temporal activity

The majority of *Culicoides* species exhibit a crepuscular peak in activity (Meiswinkel & Elbers, 2016, Mellor et al., 2000, Sanders et al., 2012); however, twilight is a period when light‐suction traps will likely exhibit decreased efficacy due to the competition between their light source acting as an attractant versus residual daylight in the environment. Trinidad's close proximity to the equator (~10° north of the equator) means that the twilight period is fairly short (~ 45 min) and consistent throughout the year, making it easier to discern any trends among *Culicoides* activity. Although the peak activity for all of the collected species occurred in the time intervals nearest sunset as expected, it was noted that a particular species, *C. aitkeni*, exhibited more specific diurnal activity, indicating a potential for light‐suction traps to underestimate or even miss these species in their collections.

This study utilized sweep netting as a collection method to facilitate a preliminary validation of the species richness collected by the static traps versus those *Culicoides* species actively flying at the study site. The sweep net sampling broadly corroborated that each of the static trap types were collecting a representative selection of the *Culicoides* species present at the site. Six of the eight species collected by the static traps were also collected using the sweep net methodology. Even though *C. furens* and *C. pusillus* were the predominant species in both the static trap and sweep net collections, the sweep net collections did highlight one area of variation in temporal activity that would warrant further investigation. Sweep net collections showed that *C. pusillus* activity peaked much earlier than *C. furens*. Additional work is required to further explore what species‐specific phototropic responses to light intensity may exist among *Culicoides* species and how this may influence any comparative assessment of their abundance in Trinidad and the greater Caribbean region.

## CONCLUSIONS

This study showed significant differences in the effectiveness of three commercially‐available static traps and a mechanical trapping method (sweep net) in relation to *Culicoides* specimen yield, sex, reproductive status and species richness. From a yield perspective, the largest number of *Culicoides* specimens were trapped with the semiochemical lure traps, followed by the UV light traps, with relatively low numbers being trapped with the incandescent light traps. Semiochemical lure traps also had the advantage of trapping very little to no by‐catch compared to the two light traps. In relation to sex, UV light traps were found to trap higher proportions of *Culicoides* males, whereas the semiochemical trap was most effective at trapping females. From a reproductive status perspective, higher percentages of blood‐fed females were collected by the sweep net method compared to the three static traps. With respect to species richness, the three static trap collections were found to be constant and comparable with the sweep furens is not a known vector species, although net collections. However, some identified biting midge species were caught exclusively by different static traps, indicating species bias to certain traps/lures. Investigations into the crepuscular activity of *Culicoides* biting midges confirmed that activity for all species identified in this study peaked during the last 15 min of daylight with certain species showing heightened activity from as early as 1 h before nightfall. It is therefore recommended that static traps are set up at least 1 h before sunset to catch as many specimens/species as possible. Overall, when planning studies involving the trapping of *Culicoides* biting midges, it is important to carefully select the trap‐type‐based around many factors including cost, length of the study, ease of sorting the trapped insects and the nature of the *Culicoides* midges being targeted. For example, in surveillance studies designed to identify the diversity of *Culicoides* species present at a particular location, multiple trapping methods should be implemented to ensure that all species are trapped, whereas studies targeting blood meal analyses should use mechanical trapping methods, such as the sweep net, which would trap more targeted blood‐fed *Culicoides* females.

## CONFLICT OF INTEREST

The authors declare no conflict of interest.

## Supporting information


**Figure S1** Pictures of some of the (*Culicoides* spp) host animals on the farm: Saanen and Anglo‐Nubian goat breeds (*Capra aegagrus hircus*). It is noteworthy that the Jarvis Dairy Goat Farm is a ‘no kill’ farm in Trinidad and Tobago that allows the animals to live out their natural lives (happily) on the farm.
**Figure S2**. Pictures of the cultivated flora found within the dairy goat farm: mulberry bushes (*Morus* spp.), trichantera shrubs (*Trichantera gigantean*), Mulato grass (*Brachiaria* spp) and Guinea grass (*Megathyrsus maximus*).
**Figure S3**. Pie charts showing the breakdown of the reproductive status (pigmented, non‐pigmented, gravid, blood fed and undetermined) by percentage (rounded to the nearest integer) of the catchment of the female Culicoides biting midges collected by each of the respective traps: A (CDC downdraft UV trap), B (CDC incandescent (white)‐light trap), C (unlit CDC trap with a semiochemical lure consisting of R‐(−)‐1‐octen‐3‐ol and CO_2_) and D (the sweep net method).
**Table S4**. Table showing total number of Culicoides species collected (male; female) by sweep net at different time points from 4:15 to 6:30 pm over four evenings of sampling during peak dry season in Trinidad.

## Data Availability

Data are available from corresponding author upon request.
