# Supplementary material for: Comparison of surveillance trapping methods to monitor Culicoides biting midge activity in Trinidad, West Indies
Source: Med Vet Entomol. 2022 Jun 15;36(4):456–68. doi: 10.1111/mve.12590 (PMC9796062; doi:10.1111/mve.12590)
Supplement: Supplementary file 1 — Figure S1 Pictures of some of the (Culicoides spp) host animals on the farm: Saanen and Anglo‐Nubian goat breeds (Capra aegagrus hircus). It is noteworthy that the Jarvis Dairy Goat Farm is a ‘no kill’ farm in Trinidad and Tobago that allows the animals to live out their natural lives (happily) on the farm. Figure S2. Pictures of the cultivated flora found within the dairy goat farm: mulberry bushes (Morus spp.), trichantera shrubs (Trichantera gigantean), Mulato grass (Brachiaria spp) and Guinea grass (Megathyrsus maximus). Figure S3. Pie charts showing the breakdown of the reproductive status (pigmented, non‐pigmented, gravid, blood fed and undetermined) by percentage (rounded to the nearest integer) of the catchment of the female Culicoides biting midges collected by each of the respective traps: A (CDC downdraft UV trap), B (CDC incandescent (white)‐light trap), C (unlit CDC trap with a semiochemical lure consisting of R‐(−)‐1‐octen‐3‐ol and CO2) and D (the sweep net method). Table S4. Table showing total number of Culicoides species collected (male; female) by sweep net at different time points from 4:15 to 6:30 pm over four evenings of sampling during peak dry season in Trinidad. [file MVE-36-456-s001.docx]

**SUPPLEMENTARY DATA**

**S1. Pictures of some of the (*Culicoides spp*) host animals on the farm:** Saanen and Anglo-Nubian goat breeds (*Capra aegagrus hircus*). It is noteworthy that the *Jarvis Dairy Goat Farm* is a “no kill” farm in Trinidad and Tobago that allows the animals to live out their natural lives (happily) on the farm.


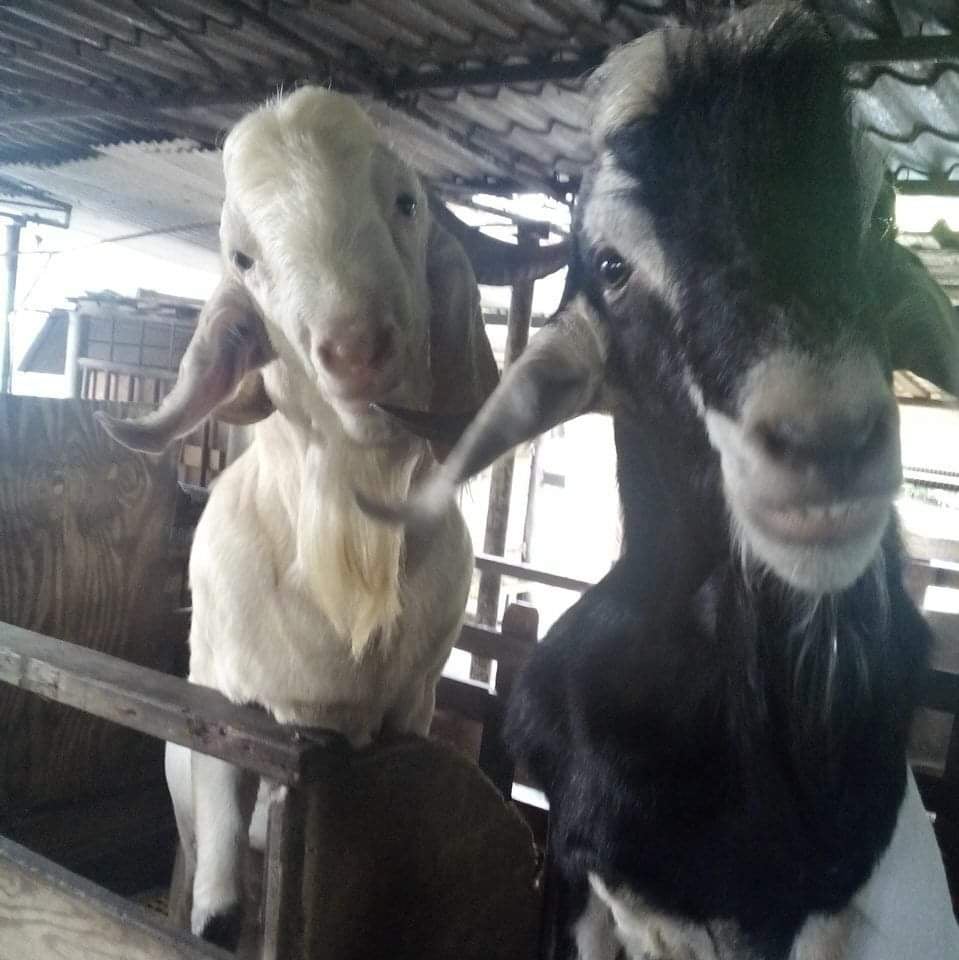

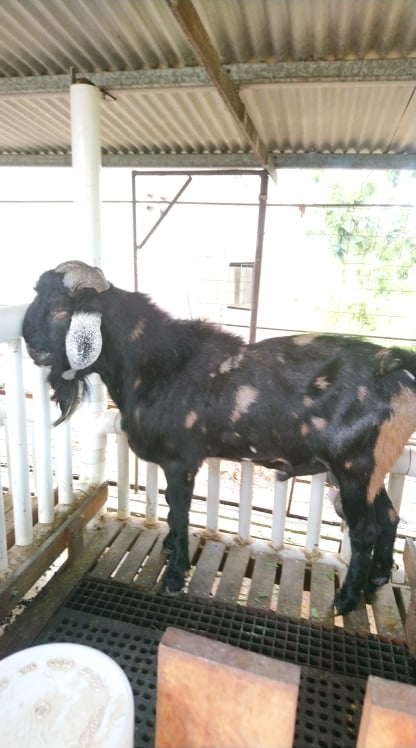


Lilly (Sanaan breed) and Chloe (Anglo-Nubian breed) Max (Anglo-Nubian breed)


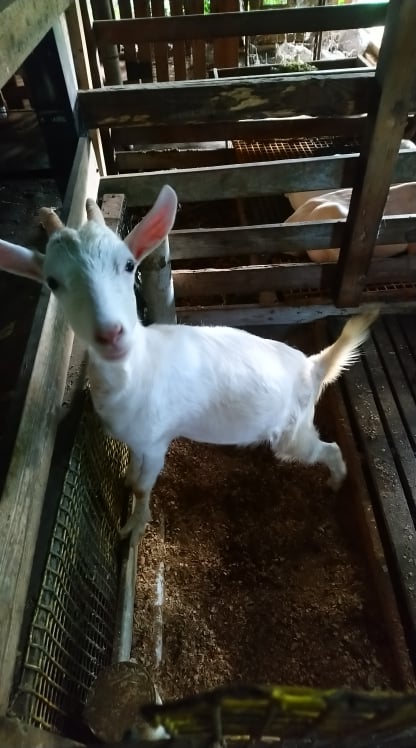

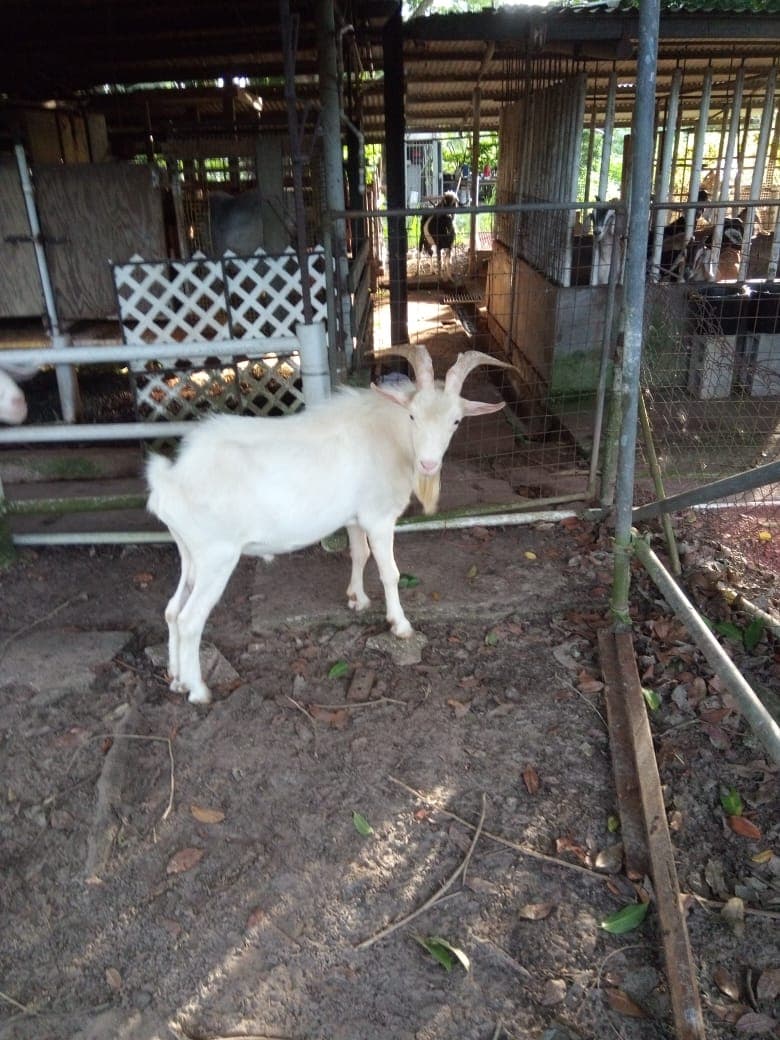


Chantilly (Sanaan breed) Lawrence (Sanaan breed)

**S2. Pictures of the cultivated flora found within the dairy goat farm:** mulberry bushes (*Morus* spp.), trichantera shrubs (*Trichantera gigantean*), Mulato grass (*Brachiaria* spp) and Guinea grass (*Megathyrsus maximus*)


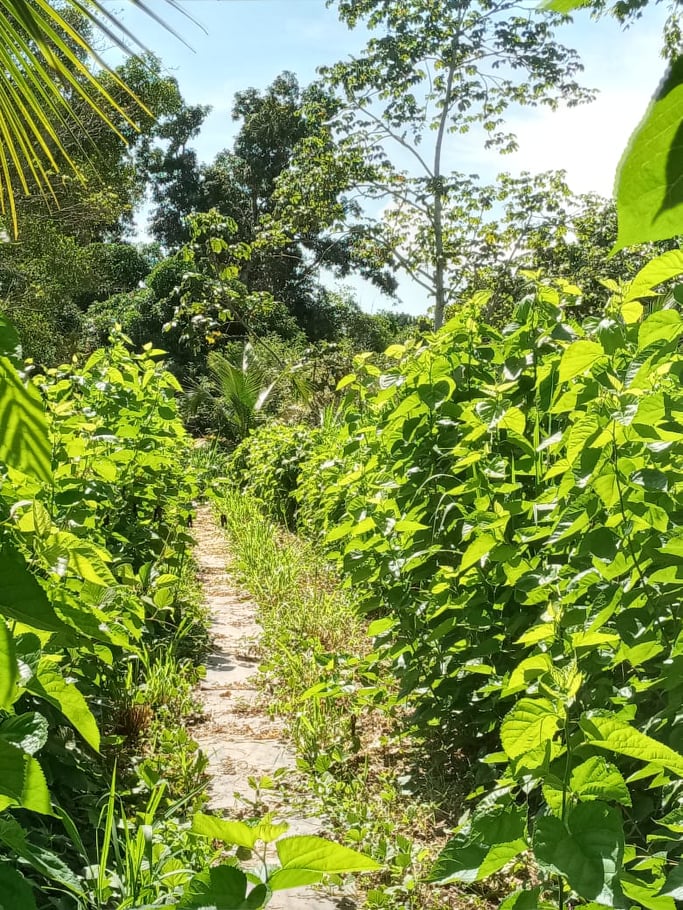

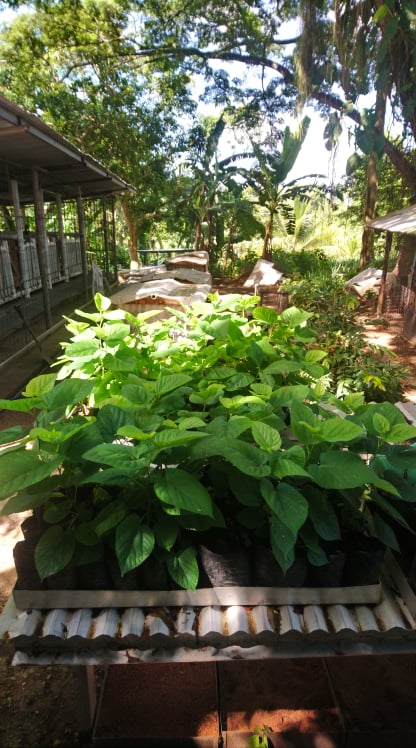


Mulberry bushes (*Morus spp.)* Trichantera shrubs (*Trichantera gigantean)*


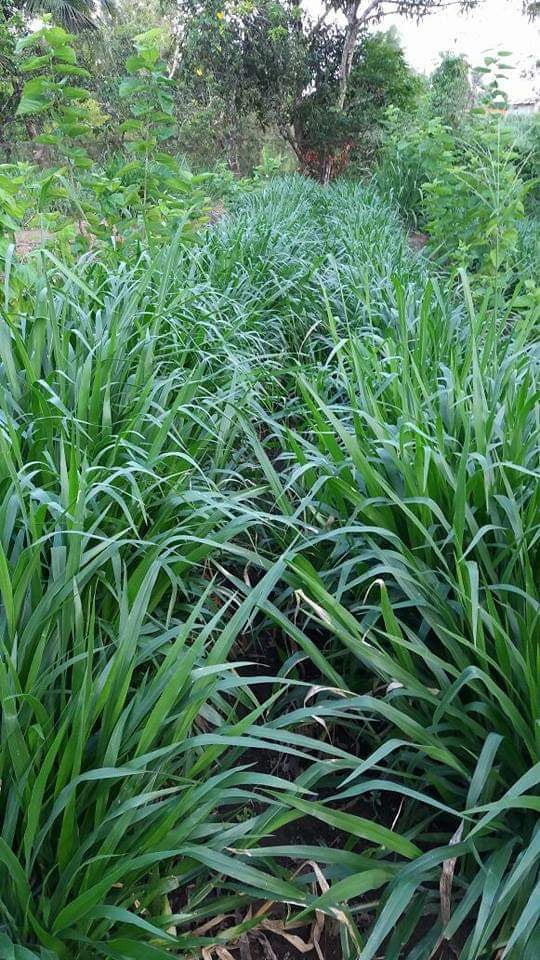

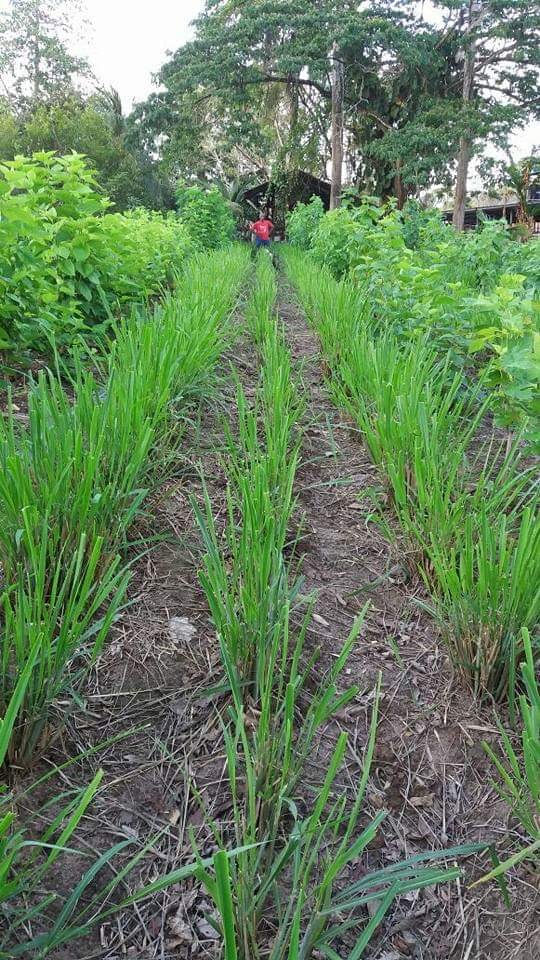


Mulato grass (*Brachiaria* spp) Guinea grass (*Megathyrsus maximus*)

**S3. Pie charts showing the breakdown of the reproductive status** (pigmented, non-pigmented, gravid, blood fed and undetermined) by percentage (rounded to the nearest integer) of the catchment of the female *Culicoides* biting midges collected by each of the respective traps: A (CDC downdraft UV trap), B (CDC incandescent (white)-light trap), C (unlit CDC trap with a semiochemical lure consisting of R-(-)-1-octen-3-ol and CO_2_) and D (the sweep net method).


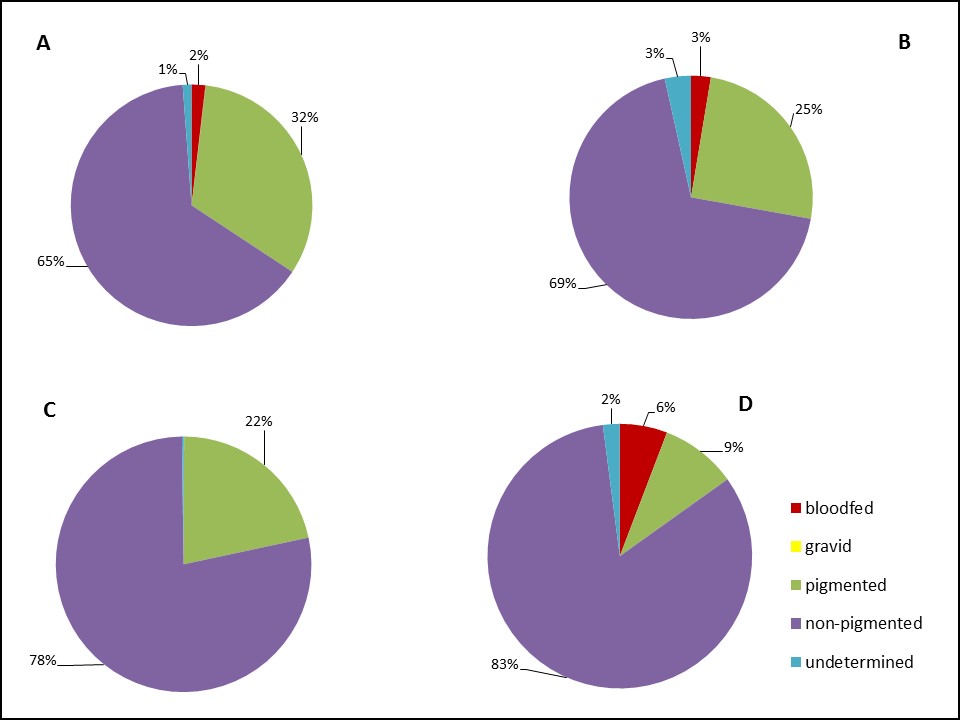


**S4.** **Table showing total number of *Culicoides* species collected (male; female) by sweep net** at different time points from 16:15 to 18:30 over four evenings of sampling during peak dry season in Trinidad.

| Collection Time Point | *Culicoides* Species | | | | | |
| --- | --- | --- | --- | --- | --- | --- |
|  | *C. aitkeni* | *C. foxi* | *C. furens* | *C. guyanensis* | *C. insignis* | *C. pusillus* |
| 16:15 | 0 | 0 | 7 (4; 3) | 0 | 0 | 2 (1; 1) |
| 16:30 | 0 | 0 | 42 (19; 23) | 0 | 0 | 0 |
| 16:45 | 0 | 0 | 22 (4; 18) | 1 (0; 1) | 0 | 5 (1; 4) |
| 17:00 | 1 (0; 1) | 1 (1; 0) | 30 (3; 27) | 0 | 0 | 13 (1; 12) |
| 17:15 | 0 | 0 | 26 (4; 22) | 0 | 0 | 5 (0; 5) |
| 17:30 | 2 (0; 2) | 0 | 52 (13; 39) | 0 | 0 | 37 (2; 35) |
| 17:45 | 4 (1; 3) | 0 | 56 (17; 39) | 0 | 0 | 31 (3; 28) |
| 18:00 | 1 (0; 1) | 0 | 80 (18; 62) | 0 | 1 (0; 1) | 15 (0; 15) |
| 18:15 | 6 (0; 6) | 1 (0; 1) | 518 (5; 513) | 0 | 1 (0; 1) | 12 (0; 12) |
| TOTAL | 14 (1; 13) | 2 (1; 1) | 833 (87; 746) | 1 (0; 1) | 2 (0; 2) | 120 (8; 112) |
